# Oxygen sensing and plant adaptation to flooding in a changing climate

**DOI:** 10.1098/rstb.2024.0238

**Published:** 2025-05-29

**Authors:** Tilo Renziehausen, Anna Dirr, Romy Schmidt-Schippers, Emily Flashman, Jos Schippers

**Affiliations:** ^1^ Bielefeld University, Bielefeld, Nordrhein-Westfalen 33615, Germany; ^2^ University of Oxford, Oxford OX1 3TA, UK; ^3^ Leibniz Institute of Plant Genetics and Crop Plant Research (IPK), Seeland, Sachsen-Anhalt 06466, Germany

**Keywords:** flooding, oxygen-sensing, plant cysteine oxidases, ERFVIIs, crop engineering

## Abstract

In times of climate change, frequency and intensity of extreme weather events are rising and thus demand a higher resilience of crop plants to environmental influences, such as flooding events, to minimize yield losses. Flooding causes acute oxygen deprivation in plants, accompanied by an energy crisis, starvation, growth retardation and ultimately increased harvest losses. At a molecular level, fluctuating oxygen concentrations are sensed via plant cysteine oxidases (PCOs) that, as part of the N-degron pathway, oxygen-dependently oxidize substrates such as vernalization 2, little zipper 2 and, most prominently, transcription factors of the group VII ETHYLENE RESPONSE FACTORS (ERFVIIs) to render them for degradation. When stabilized under hypoxia owing to the lack of oxygen, ERFVIIs act as transcriptional activators of hypoxia-response genes to evoke appropriate acclimation. Crop engineering for improved submergence resilience is an important goal for future food security, and prolonging ERFVII stability is a promising strategy. Here we discuss the potential molecular consequences of this strategy in terms of stabilization of Cys-initiating proteins in commercially relevant crop species, as well as ways in which this may be achieved, including via PCO engineering.

This article is part of the theme issue ‘Crops under stress: can we mitigate the impacts of climate change on agriculture and launch the ‘Resilience Revolution’?’.

## 1. Introduction

**Figure 1.**
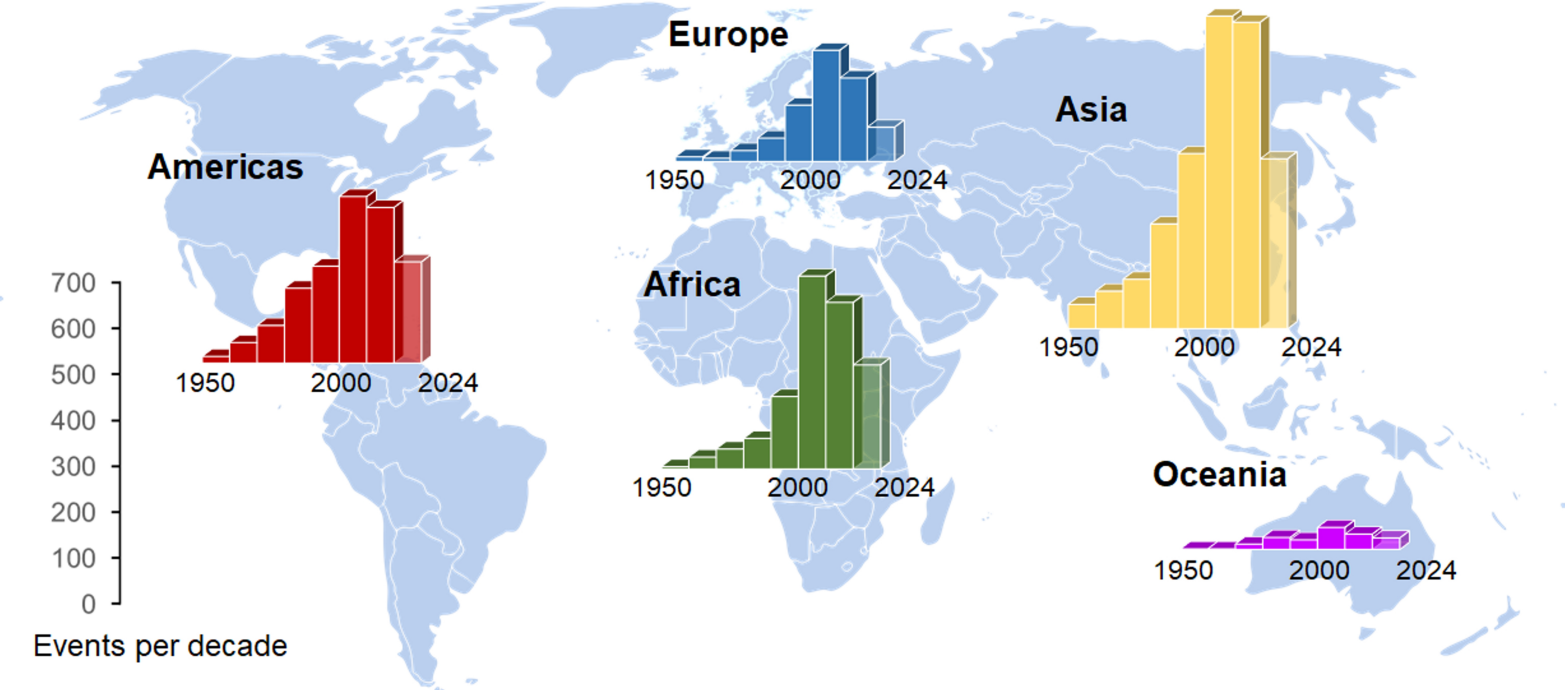
The frequency of flooding events has increased during the past decades. The figure is adapted from Bailey-Serres *et al*. [3] and is based on reported flood disaster data from the International Disaster Database from 1950 until 2024 [2]. Data are presented per decade, with the exception of the last bar representing data from 2020 until November 2024.

Human-made emissions and activities are the root cause of the current rapid climate change, resulting in a multitude of challenges [1], including securing the food supply for billions of people around the world. Of particular importance in this regard is the observed increase in the frequency of extreme weather, including long periods of drought and heat, severe storms and floods [2], all of which endanger overall agricultural productivity and cause substantial economic damage. Underlining this, the number of heavy flooding events has roughly doubled over the past 30 years (figure 1). Considering the economic damage within the agricultural sector caused by natural disasters, droughts account for the largest share in losses in crop and livestock production [4]. However, floods already represent the second most serious natural disaster, with losses amounting to US$ 21 billion, which corresponds to 19% of total losses in agriculture between 2008 and 2018 [4]. Strategies to overcome these numbers and reduce natural disaster-related crop losses for sustained food supply must therefore include endeavours to improve the resilience of essential crops towards flooding. When it comes to the mechanisms to adapt and the potential to survive flooding, there are major differences between crop plants. On the one hand, owing to its systematic submergence during cultivation, rice is commonly perceived as an example of a flooding-tolerant crop plant; nevertheless, rice harvests are also greatly impaired by adverse flooding events [5–7]. On the other hand, economically important crops such as maize, wheat and barley are very sensitive to flooding [8,9]. Thus, comparing the mechanisms by which flooding-tolerant and flooding-sensitive plants respond and adapt to flooding might be key to improving the tolerance of flooding-sensitive crops.

In this review, we provide a succinct summary of the growing threat of flooding events and the mechanisms that plants use to adapt to flooding, with a particular focus on the molecular oxygen-sensing mechanisms. Ultimately, we aim to present potential strategies to improve the flooding tolerance of important crop plants via fine-tuning oxygen sensing and the related signalling cascades.

## 2. Plant adaptation to flooding and oxygen deprivation

To secure survival during flooding events, plants have evolved various adaptations that range from adjustments at the transcript level to morphological and physiological alterations. First, the description of plant adaptations to flooding requires a distinction between possible types of flooding that a plant may face. Waterlogging is defined as the exclusive flooding of the root area, whereas flooding of the above-ground, photosynthetically active plant organs is referred to as partial or complete submergence, depending on the level up to which the plant is covered with water [10]. As a consequence, the type of flooding situation determines which acclimation responses are required to cope with the stress in the best manner. Moreover, flooding represents a multifaceted stress situation for plants that comprises many different aspects such as an energy crisis and carbohydrate starvation owing to decreased gas exchange and photosynthesis, oxidative stress caused by a reactive oxygen species (ROS) burst and altered soil chemistry [11,12]. Furthermore, flooding is immediately followed by de-submergence stress when the water recedes, posing a completely new stress situation of its own [13–15]. As a consequence, there is a variety of signalling cues, including energy signals, ROS and reactive nitrogen species, calcium (Ca^2+^), phytohormones and—most prominently—perception of the low oxygen level, that have to be dynamically integrated to achieve the optimal response [16,17].

Generally, reduced gas diffusion in water compared with air poses the most immediate impact on plants affected by flooding. This causes, on the one hand, the reduced uptake of CO_2_ and O_2_ and, on the other hand, the entrapment of the gaseous phytohormone ethylene, the latter serving as an important signalling molecule [18,19]. At the anatomical and morphological levels, plant adaptation to flooding therefore particularly involves measures to improve gas exchange with the environment and gas distribution within the plant. For example, waterlogging leads to a stronger development of adventitious roots while the growth of the main root system is inhibited [20]. Furthermore, formation of aerenchyma and radial oxygen loss barriers ensure more efficient gas transport between plant organs and reduced O_2_ loss, respectively [21–23].

As a relatively flooding-tolerant crop, rice has developed two strategies to cope with submergence. Rice plants applying the low-oxygen escape strategy (LOES) respond to submergence with rapid shoot elongation to stay above the water level and thereby enable a continued CO_2_ and O_2_ supply [24]. In contrast, the low-oxygen quiescent strategy (LOQS) is based on strict growth reduction to save energy reserves until the floods subside [24]. Together, the LOES and the LOQS illustrate the challenge for plants to balance flooding-triggered growth retardation with specific acclimation responses. This predicament applies to plants under stress in general but is of particular importance under flooding, as the reduced availability of CO_2_ and O_2_ triggers an energy crisis and starvation that demand the suppression of energy-consuming processes within the cells. In this context, limited CO_2_ uptake primarily affects the photosynthetic performance of plants, which potentially also gets restricted by comprised light availability when the flood water is murky [6]. This already indicates that a restricted CO_2_ supply is most prevalent under partial or complete submergence when photosynthetically active plant organs are covered with water. Soil waterlogging is also accompanied by decreased CO_2_ uptake owing to reduced stomatal conductance, which occurs in order to avoid dehydration symptoms in shoot tissues [25]. A lack of O_2_, however, occurs rapidly and more intensively during both waterlogging, e.g. owing to heavy rainfall, and full submergence, e.g. owing to turbid water during a flash flood. Roots in particular suffer a relatively rapid drop in oxygen concentration owing to the consumption of the remaining soil oxygen by microorganisms [19,26]. The reduction in O_2_ availability under submergence in the above-ground area, however, is not quite as severe in comparison, as plants can still exhibit residual photosynthetic activity, but this is highly dependent on the light availability under specific conditions [19].

Oxygen deprivation (hypoxia) impairs root functioning and has a direct effect on the plant’s ATP production via mitochondrial respiration: O_2_ serves as terminal electron acceptor in the mitochondrial electron transport chain and thus hypoxia considerably limits the efficiency of mitochondrial respiration and oxidative phosphorylation for ATP production, resulting in a fast and severe energy crisis [18]. Simultaneous inhibition of both photosynthesis and mitochondrial respiration forces submerged plants to tap into their carbohydrate reserves in order to augment O_2_-independent ATP production via upregulated glycolysis, which is significantly less efficient than oxidative phosphorylation [18]. Therefore, submerged plants can quickly run into carbohydrate starvation, which occurs less imminently when plants only face waterlogging and are still capable of conducting photosynthesis [12,27]. To ensure the continuation of glycolysis, flooded plants rely on ethanol and lactate fermentation for NAD^+^ regeneration, although accumulation of the products of these anaerobic pathways has negative effects on the vitality of plant cells as well, e.g. owing to the acidification of the cytosol caused by accumulation of lactate [24,28].

Taking all of this into account, enabling efficient and appropriate acclimation responses towards flooding and the associated hypoxia demands, first, a refined sensing mechanism to perceive declining O_2_ levels and, second, translation of this condition into a signalling cascade, both of which are described in the following section.

## 3. How plants sense and adjust to their cellular oxygen status

To adapt to flooding stress, the adaptive processes described above are triggered by ethylene accumulation and O_2_ depletion. A reduction in O_2_ availability is sensed by PLANT CYSTEINE OXIDASE (PCO) enzymes, which are Fe(II)-dependent dioxygenases [29]. These enzymes are kinetically tailored to act as O_2_ sensors, as their rate of activity correlates with O_2_ levels within a physiologically relevant range [30,31]. When O_2_ is available, the PCOs catalyse the dioxygenation of cysteine residues at the N-termini of their target proteins to cysteine sulfinic acid [29]; this primes their substrates for degradation via the Cys/Arg-N-degron pathway, as described below (see §3a; [29,32–34]). In low O_2_ conditions, PCO substrates remain stable.

### (a) Hypoxia acclimation via N-degron pathway-mediated control of group VII ethylene response factors stability

The Cys/Arg-N-degron pathway facilitates the degradation of N-terminally Met1–Cys2-initiating proteins where the Cys2 residue is exposed (Nt-Cys2) by methionine aminopeptidases and subsequently oxidized [35]. In plants, active PCOs catalyse the oxidation of the Nt-Cys2 residue of the ERFVIIs, which are transcription factors known to play a major role in triggering the acclimation response to flooding-induced hypoxia [32,33]. The ERFVIIs are subsequently recognized by arginyl transferases (ATE1/ATE2 in Arabidopsis), which catalyse the addition of an Arg to the ERFVII N-terminus [29], rendering them substrates for the E3 ubiquitin ligase PRT6. Subsequent poly-ubiquitination ultimately results in proteasomal degradation of the protein [35]. The auxin transport protein BIG/DARK OVEREXPRESSION OF CAB1/TRANSPORT INHIBITOR RESPONSE3 has also recently been shown to support PRT6-mediated degradation [36]. The Arg N-degron pathway is also present in mammals, where the enzymes involved have been proposed to form a super-complex in order to achieve an efficient and sequential transfer of Arg and ubiquitin [37]. However, there is no evidence for such complex formation of the plant proteins to date. Two other proteins (VERNALIZATION 2 (VRN2) and LITTLE ZIPPER 2 (ZPR2)), with roles in development, have also been identified as being regulated by PCO activity and the N-degron pathway in plants [38,39], and it is possible that other Nt-Cys2 target proteins remain to be discovered (see §4).

Reduced PCO activity in hypoxia leads to ERFVII stabilization and induction of the transcription of hypoxia-responsive genes (*HRGs*) [32–34]. ERFVII function has been characterized in detail in Arabidopsis, which contains five ERFVIIs: RELATED TO APETALA2.12 (RAP2.12), RAP2.2, RAP2.3, HYPOXIA RESPONSIVE ERF1 (HRE1) and HRE2. Their accumulation relies on their stabilization and, at least for RAP2.12, on its release from its membrane-anchor, an acyl-CoA-binding protein, triggered by a decrease in ATP [40,41]. Accumulated ERFVIIs translocate into the nucleus and induce the expression of *HRGs* involved in fermentative, carbohydrate and lipid metabolism to enable ATP production despite inhibited oxidative phosphorylation [42,43]. In the nucleus, RAP2.12, RAP2.2 and RAP2.3 are additionally stabilized through phosphorylation of a Thr residue, which has been linked to increased cytosolic Ca^2+^ levels caused by hypoxia [44]. Besides the tightly regulated stabilization and activation of ERFVIIs, their effect on the transcription of *HRGs* is also controlled by negative feedback loops. One negative feedback loop is provided by PCOs themselves, as a subset of *PCO* genes are induced by ERFVIIs [34,45]. Another negative feedback loop has been determined in the model cereal species *Brachypodium distachyon*, in which the ERF-induced BdERF961 represses the transcription of HRGs [46].

As well as PCO-mediated Cys oxidation, additional modifications to ERFVII N-termini may also be important in proteasomal signalling via the N-degron pathway. The oxidation of Nt-Cys2 and subsequent degradation of ERFVIIs has been linked to nitric oxide exposure [47], albeit this seems to be an independent mechanism from PCO-catalysed oxidation [29]. Furthermore, Nt-Cys2 sulfonic acid (–SO_3_H) formation has been found in wheat germ extracts depending on their O_2_ state [48]. While ATE accepts substrates with both Nt-Cys2–SO_2_H (sulfinic acid) and Nt-Cys2–SO_3_H [29,49], it has been suggested that another enzyme might be involved in this additional oxidation step [48]. Further investigations are necessary to elucidate the exact mechanism that produced Nt-Cys2–SO_3_H in this context.

### (b) The molecular and physiological effects of de-submergence

As floodwaters subside, de-submergence results in a rapid reintroduction of O_2_. This renewed availability of O_2_ not only marks the end of the submergence stress but also represents a novel challenge to the plant. During submergence, plant tissues have been adapted to reduced O_2_ and light conditions [14], but reoxygenation results in the production of ROS, impairment of the photosystem (photoinhibition), dehydration stress (e.g. caused by tissue desiccation and damaged roots), pre-mature senescence, stress owing to soil changes (e.g. redox potential, toxins) and nutrient deficiency [13–15,50–52]. The atypical Nt-Cys2 ERFVII transcription factor SUB1A in rice, which appears to escape N-degron-dependent degradation [33], protects plants not only during submergence but also upon reoxygenation [13]. SUB1A protects de-submerged plants against oxidative stress and dehydration stress by enabling successive closing and opening of stomata via modulated ABA sensitivity to balance CO_2_ influx to regenerate carbohydrate reserves via photosynthesis and transpiration rates to counteract dehydration [13,53]. SUB1A has also been linked to reduced chlorophyll catabolism during submergence and less damage from oxidative stress post-submergence [13,54]. These diverse functions of SUB1A indicated the involvement of ERFVII factors and the N-degron pathway in the sensing of and responding to multiple abiotic stresses. Indeed, in several plant species, a link was found to drought stress and salt stress, as well as osmotic stress [13,55–59].

Overall, the existence of a dynamic network is emerging, connecting O_2_, ethylene and ROS accumulation with the adjustment of energy and carbohydrate metabolisms to survive and recover from submergence. At the molecular level, the key determinant in establishing the proteomic landscape necessary to perform metabolic switching and activation of acclimation responses is the ability to sense O_2_ availability. In other words, PCO-controlled stability of ERFVII transcription factors and other proteins enables direct integration of the O_2_ concentration into developmental and stress adaptation responses.

## 4. Molecular targets of the oxygen-dependent N-degron pathway

**Figure 2.**
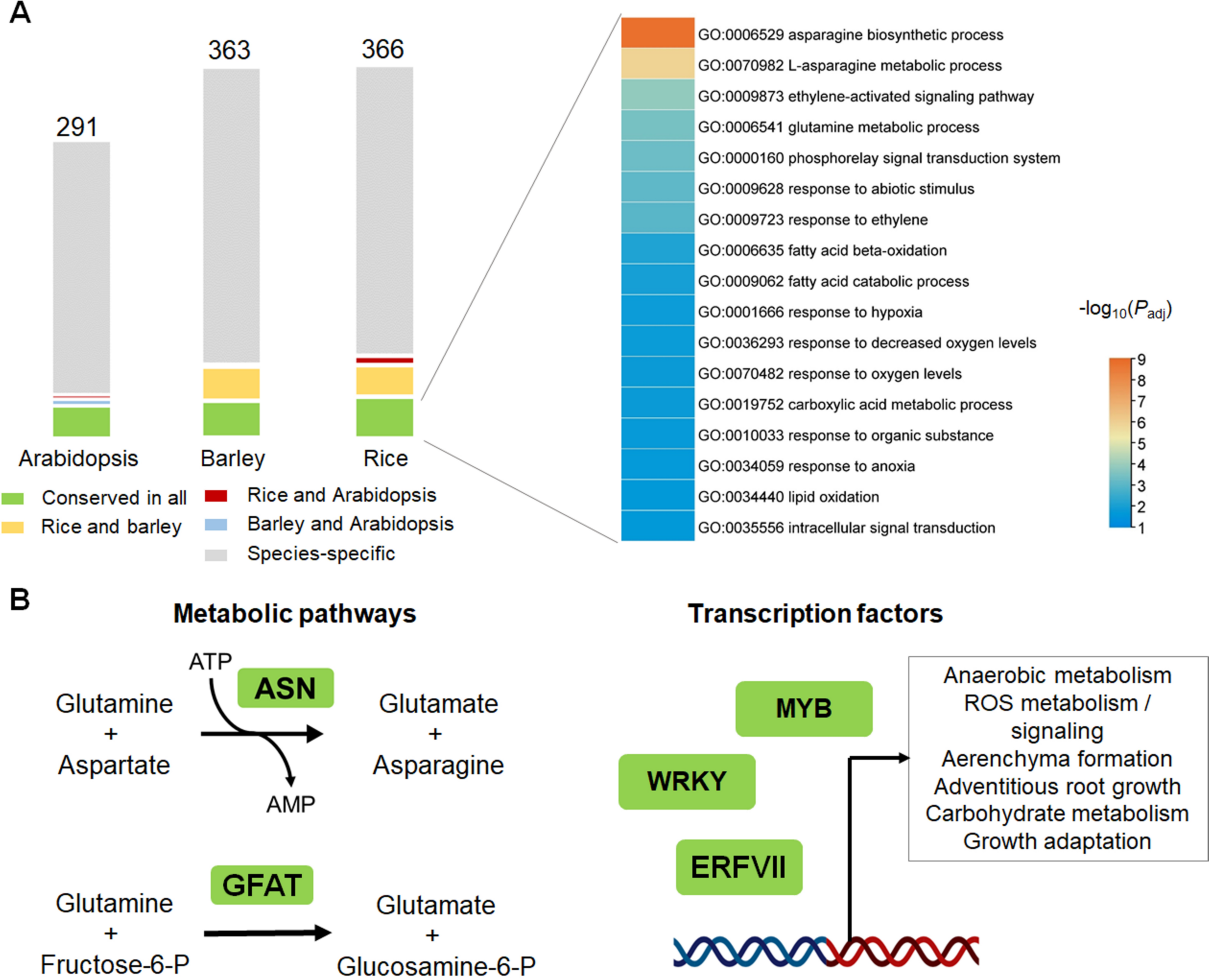
Conserved and species-specific Nt-Cys2 proteins in plants. (A) The bar chart indicates the number of genes encoding for Nt-Cys2 proteins in Arabidopsis, barley and rice. The colour code indicates conservation between the three selected species. Grey colour indicates species-specific Nt-Cys2 proteins. The right panel depicts a GO enrichment analysis of the Nt-Cys2 proteins present in all three species analysed using g:Profiler [61]. Among others, GO terms for flooding and low oxygen stress are enriched. (B) Schematic representation of conserved molecular pathways that involve Nt-Cys2 protein. On the left metabolic pathways and on the right transcriptional regulators (e.g. MYB, WRKY, and ERFVII) are highlighted.

While the impact of O_2_ on ERFVII stability is well-characterized, variable O_2_ availability is also likely to impact the stability of other Nt-Cys2 proteins via PCO activity. To better understand the impact of oxygen on molecular processes in plants, it is therefore essential to identify and validate targets of the oxygen-dependent branch of the N-degron pathway. These can be revealed by examining common and unique molecular processes in different species; we therefore assessed the conservation and divergence of the Nt-Cys2 proteome, i.e. N-terminally Met1–Cys2-initiating proteins, in *Arabidopsis thaliana* (cv. Columbia), barley (*Hordeum vulgare* cv. Morex) and rice (*Oryza sativa* cv. Nipponbare)—the latter two representing economically important crop species that highly differ in their resilience towards flooding and hypoxia. This group of proteins has previously been referred to as the MC-ome [60], whereby mainly ERFVII transcription factors have been intensely studied. It provides a resource for potential Nt-Cys2 proteins that may qualify as N-degron targets. Our analysis revealed 291, 363 and 366 loci that encode for at least one Nt-Cys2 protein in Arabidopsis, barley and rice, respectively (electronic supplementary material, table S1). Among these Nt-Cys2 proteins, 35, 37 and 42 are conserved between Arabidopsis, barley and rice, respectively (figure 2A). This group contains confirmed N-degron targets, including ERFVII transcription factors, the plant polycomb repressive complex 2 subunit VRN2 and the transcriptional regulator ZPR2 [38,39]. Still, most of the conserved potential oxygen-sensitive targets are thus far not validated. A gene ontology (GO) enrichment analysis revealed terms related to hypoxia, ethylene signalling and metabolic processes (figure 2A and electronic supplementary material, table S2). Genes belonging to these terms encode for instance ATP-dependent ASPARAGINE SYNTHETASES (ASNs), which catalyse the transfer of an amino group from glutamine to aspartate to form glutamate (figure 2B), AMP (adenosine monophosphate) and asparagine, whereby the latter serves as a nitrogen pool in plants [62]. Moreover, AMP accumulation in human cells activates AMP-activated protein kinase during nearly all mitochondrial stresses, including hypoxia [63]. Interestingly, human ASNs are Nt-Cys2 proteins and have previously been shown to be implicated in tumour survival [64], albeit they appear not to be regulated by the human PCO paralogue, 2-AMINOETHANETHIOL DIOXYGENASE (ADO) [65]. In addition, the first enzyme in the hexosamine biosynthetic pathway, glutamine : fructose-6-phosphate-amidotransferase (GFAT) is a conserved Nt-Cys2 protein. GFAT is required for the formation of UDP-N-acetylglucosamine (UDP-GlcNAc), an essential amino sugar donor for glycosylation of proteins and lipids [66], and the Nt-Cys2 feature is also found in the human orthologues of the protein. Moreover, the hexosamine pathway and the GFAT enzyme are commonly linked to tumour growth and survival in humans [67,68] and are linked to endoplasmic reticulum stress in plants [66], suggesting an intrinsic role in hypoxic adaptation. Other interesting conserved Nt-Cys2 proteins are two WRKY transcription factors (*At*WRKY14 and *At*WRKY35) implicated in the repression of thermomorphogenesis [69]. If shown to be bona fide targets, these WRKY transcription factors might accumulate to repress growth during submergence and save energy. As soon as conditions become beneficial, the oxygen-dependent breakdown would enable a rapid resumption of growth.

Between the two selected cereals, shared Nt-Cys2 proteins were identified, including an alcohol dehydrogenase (ADH; electronic supplementary material, table S1), which accumulates during low oxygen stress to promote plant survival [70]. In general, *ADH* is regulated at the transcriptional level during hypoxia through an upregulation of its expression in plants. The Nt-Cys2 feature in barley and rice might allow for a more rapid metabolic switching upon reoxygenation. In addition, both barley and rice contain a Nt-Cys2 exocyst subunit. Previously, the exocyst, a complex that controls protein trafficking to the plasma membrane, has been implicated in the regulation of root architecture during stress [71]. In the future, extensive research will be needed to validate which Nt-Cys2 proteins are indeed targets of the N-degron pathway. In particular, since excision of the primary methionine, necessary for generating the Nt-Cys2 residue for oxidation by PCOs, might not occur for all Nt-Cys2 proteins. Moreover, there are additional regulation mechanisms preventing some Nt-Cys2 proteins from being targeted by the N-degron pathway, as is the case for the ERFVII factor SUB1A in rice [33,72]. Notably, analogous to mammalian systems, there could exist additional potential targets with an N-terminally exposed Cys through the action of endopeptidases that are not reflected within the Nt-Cys2 proteome [73,74]. Nonetheless, taken together, conserved Nt-Cys2 proteins represent potential valuable targets to modulate submergence tolerance in crops.

## 5. Knowledge-based improvement of flooding tolerance in crops

The delineation of the molecular mechanisms underlying oxygen sensing and submergence tolerance in plants provides promising avenues for improving flooding tolerance in crops. The ERFVIIs themselves integrate multiple signals in response to submergence, and multiple studies have shown that stabilized ERFVIIs can support submergence survival and recovery of Arabidopsis plants [27,32,34]. Also, in rice and potentially barley, ERFVIIs are linked to tolerance to flooding [46,75]. Transient, rather than constitutive, stabilization of the ERFVIIs appears to be important for flooding tolerance, at least in Arabidopsis; in the intermediately tolerant Arabidopsis ecotype Columbia-0 (Col-0) [26], overexpressing Nt-Cys2 initiating RAP2.12 showed improved submergence survival compared to Col-0 plants overexpressing mutated RAP2.12 versions in which the Nt-Cys2 was replaced with an Ala or blocked with an N-terminal HA-tag, thus evading control by the PCOs [32]. Controlled stabilization of ERFVIIs might therefore be beneficial to both pre-adapt the plant to submergence and enhance post-submergence survival. Interestingly, small molecule PCO inhibitors, which likely compete with ERFVIIs for active site binding, can indeed prime Arabidopsis seedlings for subsequent recovery post-anoxia [76]. Moreover, screening a barley pangenome collection covering 76 barley cultivars revealed differences in the occurrence of ERFVIIs and PCOs ([77]; electronic supplementary material, table S3), suggesting variation in the PCO–ERFVII-mediated flooding adaptation response.

**Figure 3.**
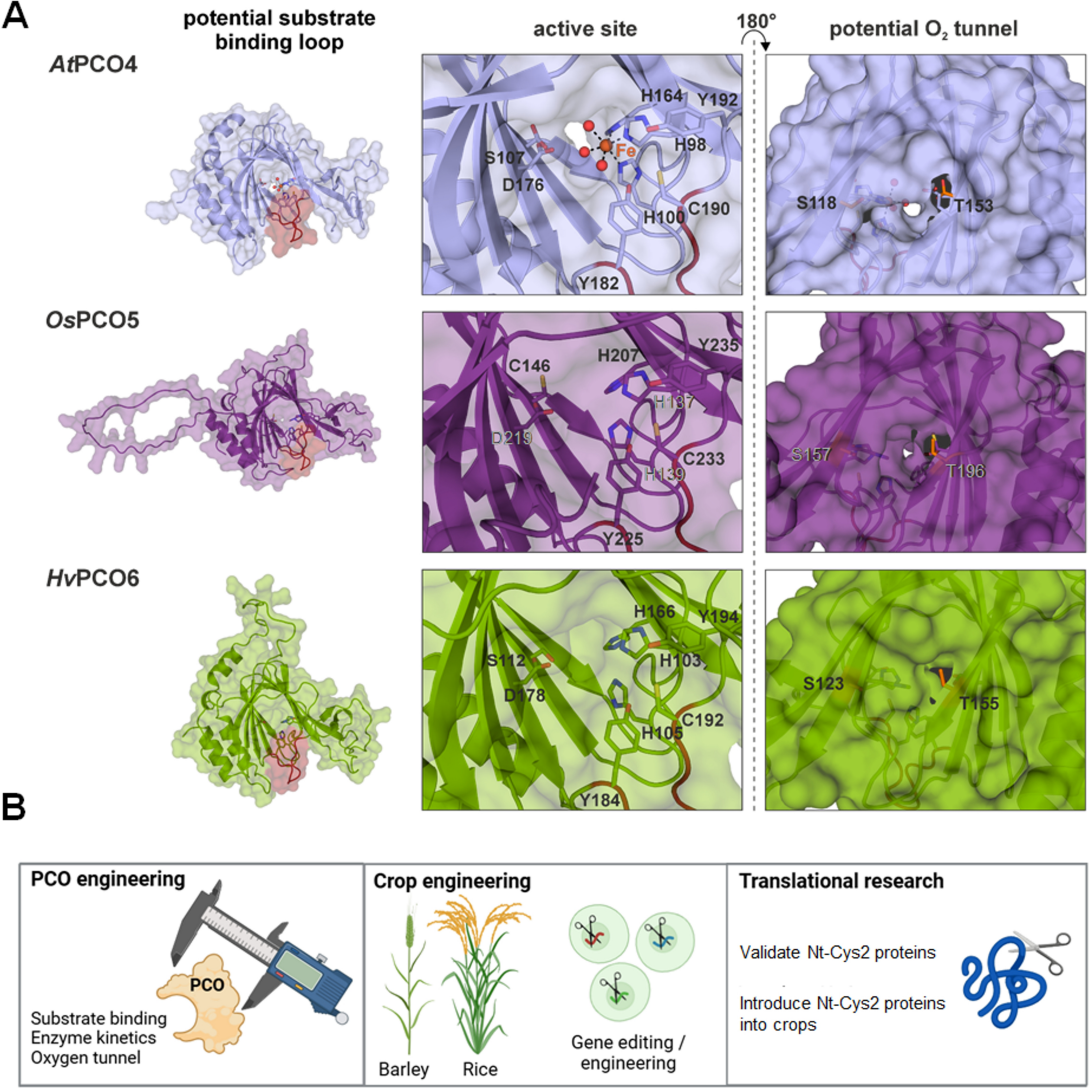
PCO engineering and improving flooding tolerance in crops. (A) Opportunities to engineer PCOs exemplified on *At*PCO4 and selected homologues. Cartoon presentation of the crystal structure of Arabidopsis *At*PCO4 (PDB: 6S7E) and the AlphaFold models of *Os*PCO5 (rice, Os05G514701) and *Hv*PCO6 (barley, HORVU.MOREX.r3.6HG0605360). The substrate-binding loop is indicated in red. In the middle, the His-triad and other conserved active site residues are shown as sticks. On the right, the orange sticks highlight residues that are at the same position as the proposed gate residues in ADO. (B) Strategies to enhance flooding tolerance through PCO engineering, crop engineering and translational research based on Nt-Cys2 proteins.

To establish plants with altered flooding tolerance, it is feasible to adopt a protein engineering strategy focusing on PCO enzymes; the PCOs are catalysts of ERFVII oxidation, therefore careful adjustment of their structure can alter their kinetic properties to modulate ERFVII stabilization. Engineered PCOs would need to retain sufficient activity to destabilize ERFVIIs under normoxia while facilitating prolonged ERFVII stability across the duration of a submergence event. One strategy to achieve this could involve reducing the affinity of PCOs for their substrates by modifying the PCO substrate-binding site, either at residues surrounding the active site or on a putative substrate-binding loop (figure 3A,B) [78]. Alternatively, it is feasible to reduce the catalytic rate of PCOs for substrate oxidation by modifying residues that facilitate aspects of catalysis such as Fe(II)–O–O activation or transition state stabilization or by decreasing their affinity for O_2_ (i.e. increasing their *K*_M_(O_2_)) [79]. The latter approach would result in a requirement for higher O_2_ levels to achieve optimal PCO activity; thus, substrates would persist at higher O_2_ levels than in the presence of wild-type enzymes (figure 3B). This could, potentially, lead to prolonged adaptation responses. A similar approach has been successfully used in the human O_2_-sensing enzyme PROLYL HYDROXYLASE 2 (PHD2) by mutating an active site residue [70]. The respective variant showed reduced O_2_ sensitivity while maintaining its primary substrate sensitivity.

Altered O_2_ sensitivity in PCOs could also be achieved by targeting engineering efforts towards a putative O_2_ tunnel that may control the rate of O_2_ delivery to the active site. Examples of enzymes with buried active sites are known to use tunnels to regulate delivery to their active site [80]. An O_2_ tunnel gated by two Cys residues has been proposed in ADO [81], and inspection of the PCO protein structures from Arabidopsis as well as a range of crop species indicates that they may also contain an O_2_ tunnel with a Ser and Thr as potential gate residues (figure 3A). Engineering the size and physicochemical properties of tunnels—in particular, bottlenecks and gates—has been successfully used to change the activity and selectivity of enzymes [82], including the oxygen-sensing PHD2, highlighting the potential for a similar successful approach with PCOs [83].

For any of these PCO engineering strategies to be successful, empirical data should ideally be obtained that demonstrates the desired effect on PCO function *in vitro* and a corresponding beneficial effect on submergence tolerance *in vivo*. Meanwhile, however, there is an urgent need to adopt successful strategies in crop plants to meet global needs. A rational, structure–function-guided approach to PCO engineering can benefit from the apparent conservation of PCO structural features across angiosperms; however, use of higher throughput synthetic biology platforms, e.g. in yeast [84], may help expedite efforts in this area. Additional complexities also arise when considering the number of PCO enzymes present in crop plants; there are five PCOs in Arabidopsis but several more in most commercial crops [45]. Special consideration will have to be given to spatial and dynamic expression and biological roles for these enzymes in order to decide whether to engineer all enzymes within a species or to target particular sub-sets. Barley, for instance, has five or six PCOs, depending on the cultivar (electronic supplementary material, table S3); this cereal therefore represents an attractive proof-of-concept model for engineering PCO activity to modulate flooding tolerance.

## 6. Outlook

Taken together, PCOs represent valuable conserved targets to fine-tune oxygen sensing and broadly modulate the response to flooding and recovery during reoxygenation. For instance, natural variation has resulted in functionally divergent isoforms with potentially different substrate specificities and kinetic activities. These differences between PCO isoforms can be exploited to fine-tune the stabilization of ERFVIIs at the onset of flooding stress responses and/or during post-flooding responses to optimize the resilience of crops to flooding. Nevertheless, it is important to ensure that engineered PCOs maintain sufficient activity during normoxia to prevent the accumulation of substrates (including putative novel substrates) and consequently their impact on metabolism and development. Hence, it is critical to establish and monitor the effect of engineered PCOs in crops to ensure yield is not impacted. Although different PCO isoforms are reported to influence the phenotypic and transcriptomic response differently [45], demonstrating the potential of tailoring isoform activities for optimal activity, more research is necessary to dissect the interplay between PCO activity, energy status, metabolic stress, development and yield in crops.

The study of the evolution of oxygen sensing in eukaryotes has revealed shared pathways and strategies in animals and plants. Still, analysis of conserved oxygen-dependent substrates between both clades is highly underexplored. For example, plant ASN and GFAT enzymes represent putative targets that in mammals play crucial roles during hypoxia survival. It would be of great interest to explore whether they, or other Nt-Cys2 initiating proteins, represent bona fide PCO targets and if modulating their levels can also affect low oxygen tolerance and survival in plants. Finally, as rice represents a rather flooding-tolerant cereal, it would be beneficial to understand if rice-specific Nt-Cys2 proteins might contribute to this enhanced fitness (figure 3B). Understanding species-specific adaptation will contribute to transferring mechanisms to other species.

Precise gene-editing techniques like CRISPR–Cas [85] have the potential to be used to incorporate base-pair changes in respective PCOs in crops, reducing time and cost for producing optimized seeds. In recent years, an increasing number of countries have allowed gene-edited food to be produced and sold (for current developments, see https://crispr-gene-editing-regs-tracker.geneticliteracyproject.org); in the past couple of years, restrictions were eased in the United Kingdom [86]. Furthermore, Europe is currently deciding on simplifying the legislation for crops that were gene-edited using new genomic techniques, e.g. CRISPR–Cas [87]. Such crops have the advantage compared to genetically modified organisms that no foreign DNA remains in the edited plant, enabling controlled optimization and potential risk reduction for farmers [87]. Early successful examples of enzyme engineering to increase the stress tolerance of crops are tomato and potato plants in which an immune protease was altered to confer resistance to late blight [88,89]. Many gene-edited plants on the current market in Asia and the United States were improved for their nutritional value, shelf-life and disease resistance (current developments: https://crispr-gene-editing-regs-tracker.geneticliteracyproject.org/). Improving agronomical plants to be more climate resilient is a new and exciting field to ensure food security in the future. Using current knowledge of molecular mechanisms underlying flooding tolerance will enable novel targeted strategies to climate-proof our precious crops.

## Data Availability

All data can be found in the electronic supplementary tables provided in addition to the manuscript document. Supplementary material is available online [90].
